# *Lactobacillus casei *modulates the inflammation-coagulation interaction in a pneumococcal pneumonia experimental model

**DOI:** 10.1186/1476-9255-6-28

**Published:** 2009-10-16

**Authors:** Cecilia Haro, Julio Villena, Hortensia Zelaya, Susana Alvarez, Graciela Agüero

**Affiliations:** 1 Instituto de Bioquímica Aplicada, Facultad de Bioquímica, Química y Farmacia, Universidad Nacional de Tucumán, Balcarce 747, CP 4000, San Miguel de Tucumán, Tucumán, Argentina; 2 Laboratorio de Bioquímica y Clínica Experimental, Centro de Referencia para Lactobacilos (CERELA-CONICET), Chacabuco 145, Tucumán, Argentina

## Abstract

**Background:**

We have previously demonstrated that *Lactobacillus casei *CRL 431 administration improved the resistance to pneumococcal infection in a mouse model.

**Methods:**

This study examined the effects of the oral administration of *Lactobacillus casei *CRL 431 (*L. casei*) on the activation of coagulation and fibrinolytic systems as well as their inhibitors during a *Streptococcus pneumoniae *infection in mice.

**Results:**

The alveolo-capillary membrane was damaged and the coagulation system was also activated by the infection. As a consequence, we could see fibrin(ogen) deposits in lung histological slices, increased levels of thrombin-antithrombin complex (TATc) in bronchoalveolar lavage (BAL) and plasma, decrease in prothrombin activity (PT) and prolonged activated partial thromboplastin time test (APTT) values. Factor VII (FVII) and factor X (FX) were decreased in plasma, whereas fibrinogen (F) and factor VIII (FVIII) were increased. The low levels of protein C (PC) in BAL and plasma proved damage on inhibitory activity. The infected animals showed reduced fibrinolytic activity, evidenced by an increase in plasminogen activation inhibitor-1 (PAI-1) in BAL and plasma. The pathogen induced an increase of TNF-α, IL-1β and IL-6 in BAL and serum a few hours after challenge followed by a significant decrease until the end of the assayed period. IL-4 and IL-10 in BAL and serum were also augmented, especially at the end of the experiment. The animals treated with *L. casei *showed an improvement of alveolo-capillary membrane, lower fibrin(ogen) deposits in lung and decrease in TATc. APTT test and PT, FVII and FX activity were normalized. L. casei group showed lower F levels than control during whole experiment. In the present study no effect of *L. casei *on the recovery of the inhibitory activity was detected. However, *L. casei *was effective in reducing PAI-1 levels in BAL and in increasing anti-inflammatory ILs concentration.

**Conclusion:**

*L. casei *proved effective to regulate coagulation activation and fibrinolysis inhibition during infection, leading to a decrease in fibrin deposits in lung. This protective effect of *L. casei *would be mediated by the induction of higher levels of IL-4 and IL-10 which could regulate the anti-inflammatory, procoagulant and antifibrinolytic effects of TNF-α, IL-1β and IL-6.

## Background

The activation of coagulation and fibrin deposition as a consequence of inflammation is well known, and can be viewed as an essential part of the host defences [1]. The hallmark of inflammatory lung diseases are fibrin deposits, which enhance the inflammatory responses by increasing vascular permeability, activating endothelial cells to produce proinflammatory mediators, and eliciting recruitment and activation of neutrophils [2]. Excessive fibrin deposition within the airways results from severe inflammation, with increased activation of coagulation, and may compromise pulmonary integrity and function [3,2].

Current evidence from human studies suggests that in lung injury there is augmented tissue factor expression, down regulation of protein C (PC), and higher plasminogen activator inhibitor -1 (PAI-1) levels. Together, these abnormalities shift the intra-alveolar environment from anticoagulant and profibrinolytic to procoagulant and antifibrinolytic [4].

The relationship between inflammation and the coagulation system is a process in which inflammation leads not only to the activation of coagulation, but coagulation also considerably affects inflammatory activity. Besides, an insufficiently controlled response can lead to a situation in which coagulation and thrombosis contribute to disease [1].

Hence, modulation of fibrin deposition through coagulation and fibrinolysis regulation may be an important therapeutic target.

Probiotic lactic acid bacteria have several inmunomodulatory effects [5,6] and anti-inflammatory properties [7,8]. Our group reported that oral administration of *Lactobacillus casei *CRL 431 to mice infected intranasally with *Streptococcus pneumoniae (S. pneumoniae) *facilitated clearance of the pathogen and modulated the inflammatory immune response with less damage to lung tissue [9].

Considering the relevant participation of the relationship inflammation-coagulation in the severity of pneumococcal pneumonia [10], the present study was conducted to examine the effects of the oral administration of *Lactobacillus casei *CRL 431 on the activation of coagulation during a *S. pneumoniae *infection in a mouse experimental model.

## Methods

### Microorganisms

*Lactobacillus casei *CRL 431 (*L. casei*) was obtained from the CERELA culture collection. It was cultured for 8 h at 37°C (final log phase) in Man-Rogosa-Sharpe broth (MRS, Oxoid), and the bacteria were harvested through centrifugation at 5,000 rpm for 10 min and then washed three times with sterile 0.01 M phosphate buffer saline (PBS), pH 7.2 [9].

Capsulated pneumococcus (serotype 14) was isolated from the respiratory tract of a patient from the Department of Clinical Bacteriology of the Niño Jesús Children's Hospital in San Miguel de Tucumán, Argentina. Pneumococci serotypification was performed in Administración Nacional de Laboratorios e Institutos de Salud-ANLIS "Dr. Malbran", Buenos Aires, Argentina.

### Animals

Six-week-old Swiss albino mice were obtained from the closed colony kept at CERELA. They were housed in plastic cages at room temperature. Each assay was performed in groups consisting of 25-30 mice (5-6 for each day before and after infection). The Ethical Committee for Animal Care at CERELA and Universidad Nacional de Tucumán approved the experiments.

### Feeding procedures

*L. casei *was administrated for 2 consecutive days at a dose of 10^9 ^cell/mouse/day [9]. *L. casei *was suspended in 5 ml of sterile 10% non-fat milk (NFM) and added to the drinking water (20% v/v). The control group received sterile NFM in the same conditions as the test group. All mice were fed a conventional balanced diet ad libitum.

### Experimental infection

*S. pneumoniae *was grown according to Racedo *et al*. [9]. At the end of the dietary treatment (on the 3rd day) the animals were challenged with the pathogen. Animals with (Lc group) and without (C group) treatment were infected by dropping 25 uL of the inoculum containing 10^6 ^CFU (log-phase) of *S. pneumoniae *in PBS into each nostril and allowing it to be inhaled. To facilitate migration of the inoculum to the alveoli, mice were held in a head-up vertical position for 2 min. Animals were sacrificed on day 0 (before infection) and at different times post-infection.

The pathogen was detected in lung and blood samples of control mice throughout the period assayed, while the group fed with *L. casei *for 2d showed a faster clearence of the *S. pneumoniae *[9]. After the challenge, we monitored the survival of mice until day 21 post-infection. All animals survived without significant differences between both groups.

### Fibrin(ogen) deposition in pulmonary tissue

Fibrin(ogen) deposition in pulmonary tissue was determined by immunohistochemical techniques. Lung samples from both groups were fixed in 4% (v/v) formalin saline solution, dehydrated, embedded in Histowax (Leica Microsystems Nussloch GmbH, Nussloch, Germany) and cut into 4 μm serial sections. For fibrin(ogen) immunostaining, lung sections were deparaffinized and endogenous peroxidase activity was quenched with a solution of methanol/0.03% H_2_O_2 _to inhibit the activity of endogenous peroxidase in the lungs (Merck, Buenos Aires, Argentina). The sections were incubated in 10% normal sheep serum and then exposed to sheep antimouse fibrinogen (purified IgG, Cedarlane, Hornby, Ontario, Canada). After washes, slides were incubated with donkey antisheep IgG peroxidase conjugate (Sigma-Aldrich Co). Peroxidase activity was detected with a 3,3'-diaminobenzidine peroxidase substrate solution (Sigma-Aldrich Co), after which a light counterstain with hematoxylin was performed [11].

### Bronchoalveolar lavage (BAL) assays

BAL samples were obtained according to the technique described previously [12]. Briefly the trachea was exposed and intubated with a catheter and 2 sequential lavages were performed in each mouse by injecting 0.5 ml of sterile PBS. The recovered fluid was centrifuged for 10 min at 900 × g. The supernatant fluid was frozen at -70°C for subsequent biochemical and haemostatic analyses.

#### Albumin content

A measure to quantitate increased permeability of the bronchoalveolar-capillarity barrier was determined colorimetrically based on albumin binding to bromocresol green using an albumin BCG diagnostic kit (Roche Diagnostics, Indianapolis, USA). The results were expressed as mg/mL.

#### LDH activity

An indicator of general cytotoxicity was determined by measuring the formation of a reduced form of nicotinamide adenine dinucleotide using Roche Diagnostic reagents and procedures (Roche Diagnostics, Indianapolis, USA). The results were expressed as U/L of BAL fluid.

### Haemostatic tests

Blood samples were obtained through cardiac puncture and were collected in a 3.2% solution of trisodium citrate at a ratio of 9:1. Plasma was obtained according to Agüero *et al *[11]. Prothrombin time (PT); activated partial thromboplastin time (APTT); factors VII, X, II, V, VIII; and fibrinogen were performed manually on fresh plasma samples. PT and coagulation factors VII, X, II and V were determined by a one-step method (Thromborel S, Behningwerke AG, Marburg, Germany). APTT and VIII were determined by mixing plasma with calcium chloride and a partial thromboplastin reagent (STA APTT, Diagnostica Stago, Asnières, France) and timing initial clot formation. Fibrinogen concentration was determined by the method of Clauss using a commercial kit and following manufacturer's instructions (Fibriprestz, Diagnostica Stago, Asnières, France) [11].

Thrombin-antithrombin complexes (TATc), markers of coagulation system activation, were determined by enzyme-linked immunosorbent assay (ELISA) technique according to manufacturer's instructions (Dade Behring, Marburg, Germany). PC and PAI-1 activities were measured by chromogenic substrate assays (COAMATE^® ^protein C, Chromogenix, Mölndal, Sweden; STACHROM^® ^PAI, Diagnostica Stago, Asnières, France). TATc, PC and PAI-1 levels were measured in BAL and plasma samples.

### Cytokines determination

Cytokines were measured in plasma and in BAL fluid; both were obtained as described above. Tumor necrosis factor (TNF-α), interleukin-1β (IL-1β), IL-4, IL-6 and IL-10 concentrations were measured with commercially available ELISA kits according to the manufacturer's recommendations (R & D Systems, MN, USA).

### Statistical analysis

Experiments were performed in triplicate (5-6 animals each time) and results were expressed as means ± SD. After verification of a normal distribution of data, 2-way ANOVA was used. Tukey's test (for pairwise comparisons of the means) was used to test for differences between the groups. Differences were considered significant at *P *< 0.05.

## Results

### Biochemical assay of BAL fluid

**Figure 1:**
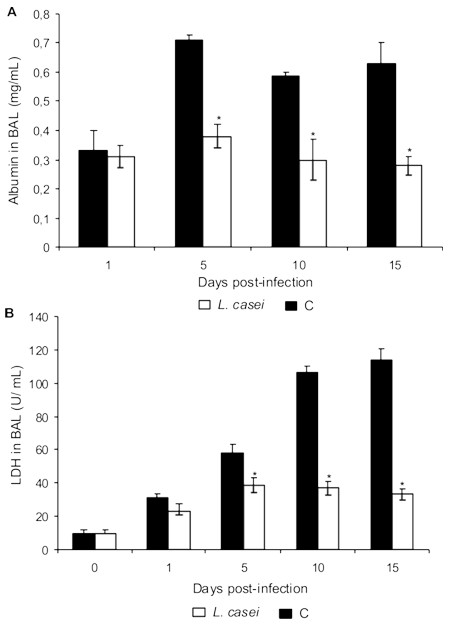
**Albumin and LDH in BAL**. *Lactobacillus casei *was orally administrated at a dose of 10^9 ^cells for 2 d before challenge with the pathogen; C group mice were infected without previous treatment. (A) Albumin content and (B) LDH activity in BAL were evaluated. Results are expressed as means ± SD (n = 5 or 6). *Significantly different from the C group and basal values (*p *< 0.05).

Albumin content and LDH activity, measured in the acellular BAL fluid, were used as indices of lung injury. Challenge with *S. pneumoniae *caused increases in BAL albumin concentration and LDH activity in both groups, but these parameters were significantly lower in *L. casei *treated mice (Figure 1).

### Fibrin(ogen) deposition in pulmonary tissue

**Figure 2:**
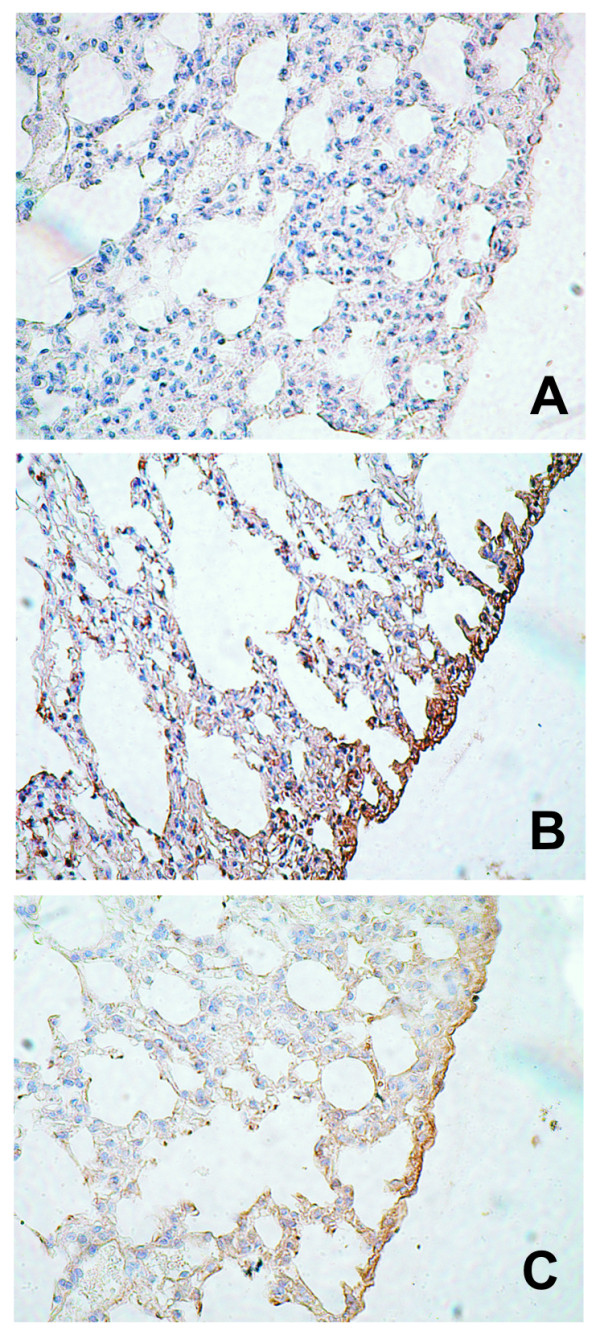
**Fibrin(ogen) deposition in pulmonary tissue**. *Lactobacillus casei *was orally administrated at a dose of 10^9 ^cells for 2 d before challenge with the pathogen; C group mice were infected without previous treatment. Panel A, C mice on d 0; Panel B, C mice on d 5 post infection; Panel C, *L casei *mice on d 5 post-infection.

Infected control animals showed fibrin(ogen) deposits in the pleura. These deposits reached their highest intensity at 10d post-infection (Figure 2). In the parenchyma, the deposits were slightly positive with a focal pattern.

The animals treated with *L casei *showed fibrin deposits of only in the pleura, with a focal pattern and lower intensity than in the C group.

### Local activation of coagulation

**Figure 3:**
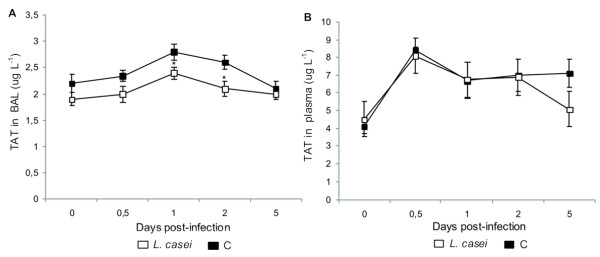
**Thrombin-antithrombin complexes (TATc)**. *Lactobacillus casei *was orally administrated at a dose of 10^9 ^cells for 2 d before challenge with the pathogen; C group mice were infected without previous treatment. (A) TATc in BAL and (B) TATc in plasma were studied. Results are expressed as means ± SD (n = 5 or 6). *Significantly different from the C group at the same time point (*p *< 0.05).

TATc levels were increased in BAL from both experimental groups, showing highest values on d 1 post-infection (Figure 3A). Then, TATc concentration decreased gradually until it reached initial values at 5 d post-infection. However, the levels of these complexes were lower in animals supplemented with *L. casei*, which remained within the normal range since d 2 post-infection.

### Systemic activation of coagulation

The increase in TATc levels in BAL was accompanied by increased systemic TATc levels since 12 h post-infection in both groups. Mice treated with *L. casei*, returned to normal values on d 5 after challenge, whereas the control group continued with higher values (Figure 3B).

**Figure 4:**
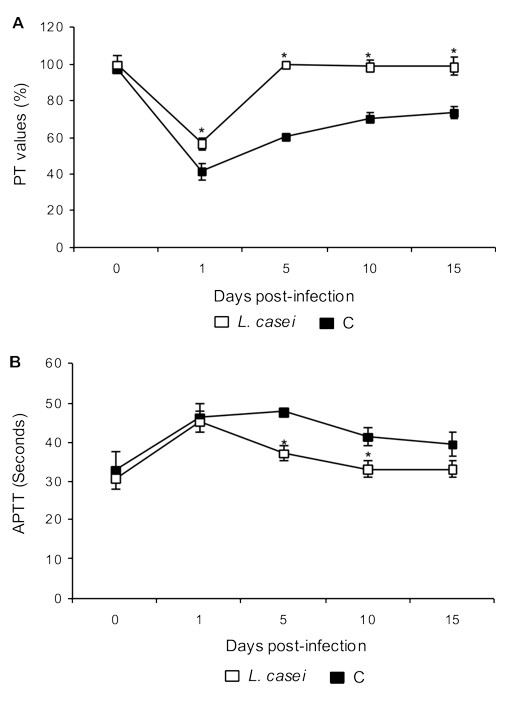
**Prothrombin time and activated partial thromboplastin time**. *Lactobacillus casei *was orally administrated at a dose of 10^9 ^cells for 2 d before challenge with the pathogen; C group were infected without previous treatment. (A) Prothrombin time and (B) activated partial thromboplastin time were studied. Results are expressed as means ± SD (n = 5 or 6). *Significantly different from the C group (*p *< 0.05).

The percentage of prothrombin activity decreased on d 1 post-challenge in both experimental groups. However, values were significantly lower in the control mice (Figure 4A). The *L casei *treated mice showed normal PT values since d 5 post-infection, whereas the control group did not reach normal values at any of the assessed periods.

After infection, APTT values were prolonged in both experimental groups (Figure 4B). The mice supplemented with *L. casei *normalized this parameter on d 5 post-infection, whereas the control group did so only on d 10 post-infection.

### Coagulation factors

**Figure 5:**
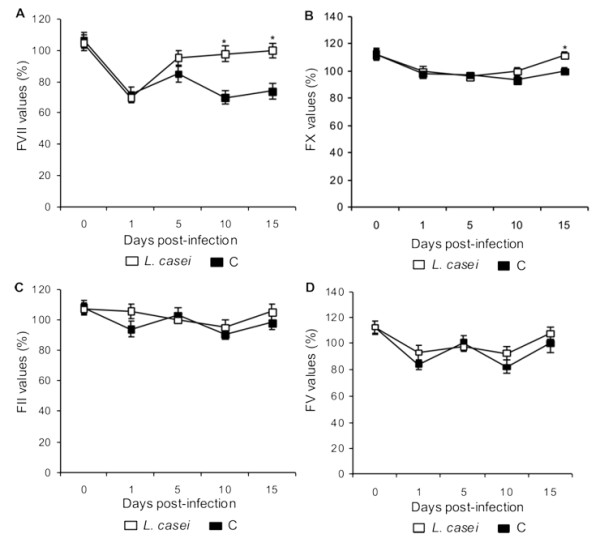
**Coagulation factors**. *Lactobacillus casei *was orally administrated at a dose of 10^9 ^cells for 2 d before challenge with the pathogen; C group were infected without previous treatment. (A) Factor VII, (B) factor X, (C) factor II and (D) factor V activities were studied. Results are expressed as means ± SD (n = 5 or 6). *Significantly different from the C group (*p *< 0.05).

FVII concentrations decreased in both groups after the challenge, reaching minimum levels on d 1 post-infection. Only mice treated with *L. casei *normalized the FVII values since d 5 post-infection (Figure 5A). These results showed a similar behaviour to the prothrombin activity described above.

FX values decreased in both groups after the infection, although the animals supplemented with *L. casei *could normalize this parameter on d 15 post-infection (Figure 5B). The C group showed lower values until the end of the experiment.

No differences between groups were found in the levels of FII during the studied period (Figure 5C).

The FV levels showed normal values during the whole assayed period in both experimental groups (Figure 5D). These results would indicate that liver functionality was preserved.

**Figure 6:**
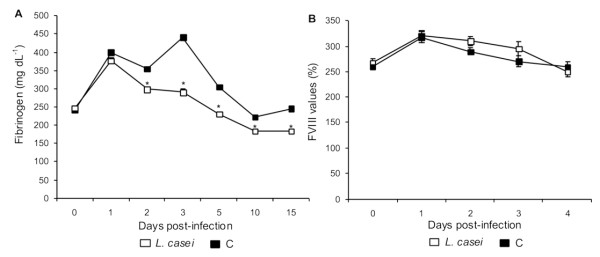
**Fibrinogen levels and factor VIII activity**. *Lactobacillus casei *was orally administrated at a dose of 10^9 ^cells for 2 d before challenge with the pathogen; C group were infected without previous treatment. (A) Fibrinogen levels and (B) factor VIII activity were studied. Results are expressed as means ± SD (n = 5 or 6). *Significantly different from the C group (*p *< 0.05).

The infection caused an increase in fibrinogen concentration in both groups on d 1 post-infection. After that, the animals supplemented with *L casei *showed lower concentrations (*p *< 0.05) than control mice until the end of the experiment (Figure 6A). The *L. casei *group returned to nomal values on d 5 post-infection, while the C group did so on d 10 post-infection.

Both groups showed increased FVIII levels after challenge (Figure 6B). The peak was reached on d 1 post-infection, and then levels dropped and returned to baseline within d 4 post-infection with no differences between control and treated groups.

### Coagulation regulators in blood and lungs

**Figure 7:**
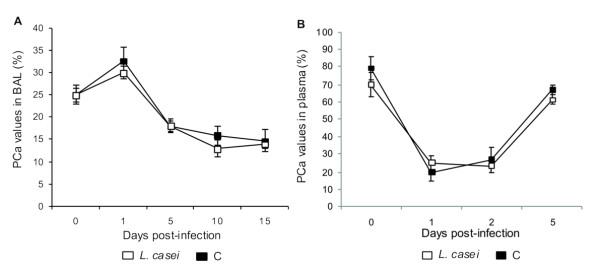
**Protein C activated (PC)**. *Lactobacillus casei *was orally administrated at a dose of 10^9 ^cells for 2 d before challenge with the pathogen; C group were infected without previous treatment. (A) PC in BAL and (B) PC in plasma levels were studied. Results are expressed as means ± SD (n = 5 or 6). *Significantly different from the C group (*p *< 0.05).

The PC system provides important coagulation control. We studied the levels of PCa in BAL and in plasma to evalutate the anticoagulant activity during the infection. After challenge with *S. pneumoniae*, PCa increased in BAL in both groups, reaching a peak on d 1 post-infection (Figure 7A). After that, the values dropped and remained decreased until d 15 post-infection. No significantly differences between control and treated groups were found throughout the studied period. The PCa values in plasma showed a different kinetic to the one described in BAL (Figure 7B). The infection induced a significant decrease in the plasma levels of PCa on d 1 post-infection in both groups, returning to baseline on d 5 post-infection.

**Figure 8:**
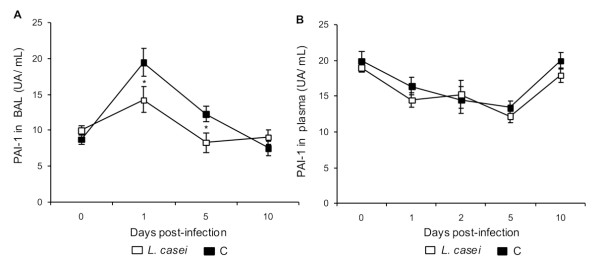
**Plasminogen activator inhibitor-1 (PAI-1)**. *Lactobacillus casei *was orally administrated at a dose of 10^9 ^cells for 2 d before challenge with the pathogen; C group were infected without previous treatment. (A) PAI-1 in BAL and (B) PAI-1 in plasma activity were studied. Results are expressed as means ± SD (n = 5 or 6). *Significantly different from the C group (*p *< 0.05).

Levels of PAI-1 in BAL increased after infection in both experimental groups, reaching a maximum on d 1 post challenge (Figure 8A). However, the mice treated with *L. case*i had significantly lower values than the control group. The PAI-1 values returned to baseline in the treated group sooner (d 5) than in the control (d 10).

Systemic PAI-1 levels showed a similar decrease in both groups, reaching normal values on d 10 post-infection (Figure 8B).

### Cytokines

**Figure 9:**
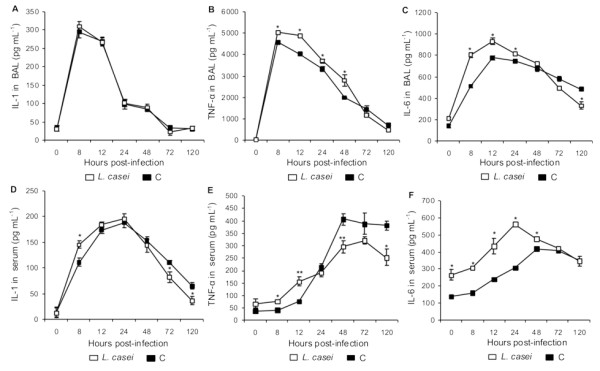
**IL-1β in BAL (A) and in serum (D); TNF-α in BAL(B) and in serum (E); IL-6 in BAL (C) and in serum (F) of mice fed *L. casei *for 2 d before (d0) and after challenge (d 1, 5, 10 y 15) with *S. pneumoniae***. Control mice were challenged with the pathogen without previous treatment. Results are expressed as means ± SD (n = 5 or 6). Asterisks represent significant differences from the C group at the same time point (*p < 0.05, **p < 0.01).

The levels of TNF-α, IL-1β and IL-6 in BAL before infection were similar in both groups. After challenge with the pathogen, these cytokines increased significantly, reaching a peak between 8 h and 12 h post-infection with higher values of TNF-α and IL-6 in the *L. casei *group. Afterwards, the values of TNF-α and IL-1β decreased gradually until they returned to base levels on d 5, whereas IL-6 concentration remained elevated with significantly higher values in the control group (Figure 9).

The serum levels of TNF-α, IL-1β and IL-6 augmented after challenge, reaching the maximum values between 24 h and 48 h post-infection. However, mice supplemented with *L. casei *showed lower levels of TNF-α and IL-1β than the control group on d 5 post-infection. Treatment with *L casei *induced a stronger increase in IL-6, with values higher than those in the control group until 48 h post-infection. After that both groups showed similar values.

**Figure 10:**
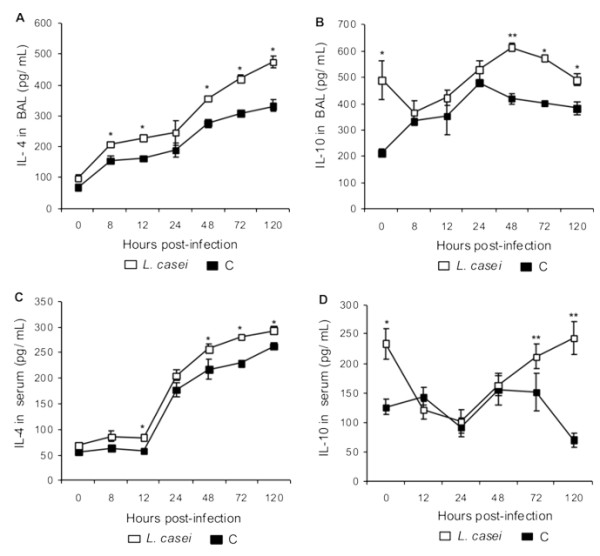
**IL-4 in BAL (A) and in serum (C); IL-10 in BAL(B) and in serum (D) of mice fed *L. casei *for 2 d before (d0) and after challenge (d 1, 5, 10 y 15) with *S. pneumoniae***. Control mice were challenged with the pathogen without previous treatment. Results are expressed as means ± SD (n = 5 or 6). Asterisks represent significant differences from the C group at the same time point (*p < 0.05, **p < 0.005).

The infection induced a progressive increase in the levels of IL-4 in BAL and in serum in both experimental groups; however, IL-4 values in the *L. casei *mice were significantly higher than those in the control group (Figure 10).

Treatment with *L. casei *enhanced the levels of IL-10 in BAL and in serum prior to infection (Figure 10). After 8 h post-challenge, both groups showed a progressive increase in IL-10 in BAL, which remained high up to d 5 post-infection. In the *L. casei *group, IL-10 in BAL was significantly higher than in the control group since d 2 post-infection. The values of serum IL-10 in the *L. casei *group were higher than in the control group on d 3 and 5 post-infection.

## Discussion

Even though the inflammatory response and coagulation activation exert an obvious protective function, the uncotrolled functioning of these processes would be harmful for the host.

Bearing in mind the previous experiences of our work team concerning the ability of *L casei *to modulate the immune response and protect mice against infection by *S. pneumoniae *[9], we decided to investigate whether this probiotic lactic acid bacteria could also regulate the haemostatic processes during pneumonia and prevent excessive fibrin formation [1], which increases the inflammatory response even more [2].

In order to find out the intensity of the damage induced by the pathogen at the lung level, we determined albumin concentration and LDH activity in BAL [13]. We observed that the *S. pneumoniae *induced increase in albumin concentration and in LDH activity in both groups, however these alterations were significantly smaller in *L. casei *treated mice. These results would indicate lower tissue damage and improvement in the permeability of the alveolo capilar membrane. In adition, the supplemented animals showed lower deposits of fibrin in lung. This result would be evidence for the inflammatory response modulation [2].

To known the procoagulante state in lung, it was determined the levels of TATc in BAL. This marker was increased in both groups on 1 d post-infection, but the levels of these complexes were lower in animals supplemented with *L. casei *and remained within the normal range since d 2 post-infection.

In order to study the procoagulante state at systemic level we also determined TATc in plasma. The results evidenced activation of the coagulation system in both groups since 12 h post-infection. Only the *L. casei *group reached de normal values on d 5 after challenge.

On the basis of the fact that *L. casei *was able to regulate fibrin deposition in lung during infection, we continued to study its effects on different hemostatic plasmatic parameters using our experimental model.

Considering that coagulation activation in lung is predominantly mediated by the extrinsic pathway, we investigated the possible alteration in prothrombin activity. We observed that the pathogen induced a decrease in prothrombin activity since d 1 post-infection in both experimental groups. Similar findings were reported by Reitsma *et al*. [14]. This behaviour could be attributed to the consumption of coagulation factors of the extrinsic pathway by its activation at the pulmonary level. This activation is probable due the greater expression of FT induced by TNF-α and IL-6 [10,15] whose level in serum and BAL were singnificantly increased between 8 and 48 h post-infection. The early increase of TNF-α is required to an adequate antibacterial response at an infection site [16]. Consequently, regulation of the inflammatory response by anti-inflammatory cytokines is essential to prevents damage to the host.

The animals that received *L. casei *recovered and finally normalized the prothrombin activity in plasma on d 5 post-infection, while the control animals recovered partially this parameter. This different behavior could be a consequence of the effect of *L. casei *on cytokines release [17]. Mice treated with *L. casei *showed lower serum levels of TNF-α and IL-1 between d 3 and 5 after challenge. At the same time the treated animals showed higher levels of IL-10 and IL-4. This increase could help to reduce the production of pro-inflammatory cytokines and prevent excessive expression of FT [18-21].

The study of plasmatic levels of the coagulation factors showed that FVII and FX followed a similar kinetic than PT. Reitsma *et al*. [14] also observed a decrease in FVII and FX in an endotoxemia model. We found that *L. casei *was effective to normalize the activity of these coagulations proteins. The beneficial effect of the lactic acid bacteria could also be due to the balance between pro and anti-inflammatory cytokines.

The levels of FII and FV were not significantly altered by the infection, probably due to their longer half-life and to the characteristics of the experimental model used.

In the present study we observed that infection induced prolongation of the APTT test, probably because of the thrombin generated by the extrinsic pathway. However, the animals treated with *L. casei *reached normal values earlier than the C.

On the basis of the hypothesis suggested by Reisman *et al*. about the fact that high plasma levels of coagulation proteins might reflect an inflammatory reaction, in this study we performed determinations of FVIII and fibrinogen. The infection induced an increase in FVIII during the first few hours after its induction, reaching a maximum value at 24 h. Reitsma *et al*. also reported an increase in FVIII activity in an model of endotoxemia [14]. In the present work, we could not see any effect of *L casei *on FVIII plasma activity, possibly because of that the changes are produced in few hours after infection.

Fibrinogen is another coagulation factor commonly used as an acute phase protein. We found that the infection induced increase in fibrinogen since d 1 post-infection, an effect that was regulated when *L. casei *was administered. Similar result was reported with a functional food product containing *L. plantarum *299 v [22,23].

The activation of the coagulation mechanism during a severe inflammatory process leads to a consumption of its inhibitors in an attempt to control such activation. In this process, the protein C system is altered, decreased plasma levels being detected [24] as a consequence of its consumption and decreased liver synthesis [1]. Besides, thrombomodulin, the main PC co-factor, has been proved to decrease its expression on endotelial cells due to the action of cytokines such as TNF-α e IL-1β, leading to a dysfunction in this system [10]. In our infection model, PCa remained decreased in plasma and BAL during most of the period studied, which would indicate that the inflammatory response effectively damages this coagulation control system. In the present study no recovery in PCa levels by *L. casei *administration was observed.

Hemostasis is further controlled by the fibrinolytic system, which degrade fibrin clots. The main inhibitor of the plaminogen activators is PAI-1, which is produced by the endothelium and the liver and increase in PAI-1 levels are induced by TNF-α and IL-1β [25]. Thus, inhibition of the fibrinolytic system is another event that facilitates fibrin deposition. This inhibition might result from the increase in pro-inflammatory cytokines [26]. Challenge with *S. pneumoniae *increased significantly the values of PAI-1 en BAL, leading to the local inhibition of fibrinolysis in the lungs during the infection. However, *L. casei *treated mice showed a less pronounced increase in PAI-1 in lung. This lower inhibition of local fibrinolysis could account for the fewer fibrinogen deposits observed in lung in this group.

The antiinflamatory effect of certain probiotic strains is achieved though the induction of immunoregulatory cytokines such as TGF-β, IL-10 and IL-4. The *L. casei *group showed levels of IL-10 and IL-4 in BAL and serum significantly higher that those in the control group during the late stage of the infection. This difference could be responsible for the protective effect of the lactic acid bacterium since IL-10 inhibits the synthesis of pro-inflammatory cytokines such as TNF-α and IL-1 *in vitro *[27,28] and attenuate the increase in PAI-1 concentrations during human endotoxemia [29]. IL-4 had no significant effect on PAI-1 production but can regulate the pro-coagulant activity [19].

## In conclusion

we showed that the preventive administration of *L. casei *was effective to regulate coagulation activation and fibrinolysis inhibition during the infection, which led to a decrease in fibrin deposits in lung. This protective effect of *L. casei *would be mediated by the induction of higher levels of anti-inflammatory interleukins such as in IL-4 and IL-10, which were observed in our experimental model. These interleukins would contribute to regulate the proinflammatory, procoagulant and antifibrinolytic effects of TNF-α, IL-1β and IL-6.

This new line of research opens novel posibilities for the application of probiotics in the prevention of pathologies in which the inflammation-coagulation interaction plays a major role. Diseases associated with high levels of PAI-1 such as cardiovascular disease or acute lung injury and acute respiratory distress syndrome could be an appropriate target. It is hoped that the knowledge gained in unraveling the pathophysiology of coagulation and inflammation will result in further refinements and improved therapies for patients with severe systemic injuries and septic shock.

## Abbreviations

APTT: activated partial thromboplastin time; BAL: bronchoalveolar lavage; IL: interleukin; *L. case, Lactobacillus casei *CRL 431; NFM: non-fat milk; PAI-1: plasminogen activator inhibitor -1; PBS: phosphate buffer saline; PC: protein C; PCa: activated protein C; PT: prothrombin time; *S. pneumonie, Streptococcus pneumonie*; TATc: thrombin-antithrombin complexes; TNF-α: tumor necrosis factor alpha.

## Competing interests

There are non-financial competing interests (political, personal, religious, ideological, academic, intellectual, commercial, or any other) to declare in relation to this manuscript.

## Authors' contributions

CH did the experimental work, the data analysis and prepared the manuscript; JV contributed to the drafting of the paper; HZ contributed with the experimental work; SA contributed with the designs of study; GA revised the manuscript for the intellectual content and gave final approval. All authours have read and approved the final version of the manuscript. 
